# Serum separator tube method for matrix-assisted laser desorption/ionization time-of-flight analysis

**DOI:** 10.1099/acmi.0.000011

**Published:** 2019-04-16

**Authors:** Sarit Freimann, Maanit Shapira, Abed Athamna

**Affiliations:** 1 Clinical Microbiology Laboratory, Hillel Yaffe Medical Center, Hadera, Israel; 2 Laboratory Division, Hillel Yaffe Medical Center, Hadera, Israel

**Keywords:** rapid identification, MALDI-ToF MS, blood culture, differential centrifugation, serum separator tube

## Abstract

**Background:**

Without appropriate treatment, bloodstream infections have a high mortality rate. Quicker identification of the microbial pathogen allows the clinician to develop an initial strategy of antimicrobial therapy. Sample preparation protocols for matrix-assisted laser desorption/ionization time-of-flight mass spectrometry (MALDI-ToF MS; Bruker Daltonics for Microflex LT spectrometer) technology were evaluated in an attempt to identify pathogens directly from positive blood culture bottles and thus shorten the time to identify them. This application requires preparatory processing because blood culture bottles contain undesirable proteins. This study aimed to evaluate two methods for microbial preparation for identification by MALDI-ToF MS.

**Methods:**

This study evaluated two methods for microbial preparation from 200 positive blood culture samples, half prepared by the differential centrifugation method and half with the serum separator tube method for identification by MALDI-ToF MS. Both methods were compared to conventional methods such as VITEK II and ChromAgar culture plates.

**Results:**

All Gram-negative bacteria tested were identified correctly by MALDI-ToF MS compared to conventional methods, regardless of the preparation method. However, more Gram-positive bacteria were identified when the serum separator tube method was used (83.3%) compared with the differential centrifugation method (65.3  %). Moreover, the serum separator tube protocol requires 12–15 min, while the differential centrifugation protocol requires 30–45 min.

**Conclusions:**

Sample preparation using the serum separator tube method is easy to perform, fast and reliable for accurate microbial identification by MALDI-ToF MS technology.

## Introduction

Blood stream infections (BSIs) are associated with high rates of morbidity and mortality if untreated [1]. Delay in diagnosing the causative pathogenic agent can result in empirical treatment with broad-spectrum antimicrobial agents. This intervention risks the development of antimicrobial-resistant pathogens. Therefore, early initiation of appropriate antimicrobial therapy is a key factor in the outcome of BSIs and prevention of antimicrobial-resistant bacteria [2–4].

Conventional methods for identifying micro-organisms are time-consuming. The positive blood cultures are Gram-stained and sub-cultured on solid medium, and incubated for 18–24 h. This is followed by biochemical tests or the use of an automated system such as Vitek II, in addition to the other confirmatory tests that are needed to identify the causative organism.

Fast identification of micro-organisms directly from positive blood cultures provides the clinician with information to select the appropriate antimicrobial agent, along with data regarding intrinsic resistance, which can reduce mortality [5]. Moreover, prompt prediction of antimicrobial resistance ensures the best available treatment for the patient in order to avoid the use of broad-spectrum therapy ,which may lead to the development of antimicrobial-resistant pathogens. In the last two decades, matrix-assisted laser desorption/ionization time-of-flight mass spectrometry (MALDI-ToF MS) has become routine for microbial identification and use in diagnostic microbiology [6]. Microbial identification by MALDI-ToF MS is based on analysis of the proteic spectrum of the micro-organism. Attempts have been made to identify micro-organisms directly from positive blood cultures using MALDI-ToF MS.

Numerous protein extraction methods have been described for direct identification of pathogens from positive blood culture samples for MALDI-ToF MS [7–10]. These methods were designed to prepare microbial sediment, free from human blood cells, proteins and culture medium, which may interfere with identification. The process involves detergent lyses of the human cells, followed by centrifugation and washing of the bacterial pellet using a commercial kit or various other reagents, such as saponin and a sodium dodecyl sulphate (SDS) solution [11–15]. Other methods have utilized serum separator tubes (SSTs) [11, 16–18], differential centrifugation (DC) [8, 19–22] or ammonium chloride lysis [23–26]. Some of these methods are expensive and can vary in preparation time and accuracy. The present study compared the results of the DC and SST methods for preparing microbial samples from blood cultures and assessed the resulting MALDI-ToF MS identification accuracy.

## Methods

### Blood cultures

This study was performed at the Clinical Microbiology Laboratory of Hillel Yaffe Medical Center, Hadera, Israel. Blood culture samples were collected at bedside and inoculated directly into BD BACTEC culture bottles (Becton Dickinson, Franklin Lakes, NJ, USA). They were incubated in the automated BD BACTEC FX system (Becton Dickinson, Franklin Lakes, NJ, USA) for up 5 days. When the blood culture bottles signalled positive (indicating microbial growth) they were removed from the instrument and aliquots of the blood culture were Gram-stained and subjected to subculture for routine conventional identification. At the same time, aliquots were taken from positive blood culture bottles and subjected to two methods for preparing bacterial protein extract and recovering micro-organisms for identification using MALDI-ToF MS.

### Conventional identification

Following Gram-staining, sub-culturing on various solid media was used, including ChromAgar plates (Hylabs, Israel). These plates utilize a chromogen mix that consists of artificial substrates (chromogens) that release different coloured compounds upon degradation by specific microbial enzymes, thus allowing direct identification of certain species of micro-organisms, as previously described [27].

The isolated colonies were identified using VITEK II software, version 05.04 (Advanced Expert System software, version 1.9.0; Biomerieux, Marcy Etoile, France). The cards used for identification were assessed monthly for quality control [13]. A 16S rRNA was performed for some cases when identification by ChromAgar plates and VITEK II was difficult.

### Sample preparation for micro-organism identification with MALDI-ToF MS

Samples from positive blood cultures were prepared for MALDI-ToF MS identification using the DC and the SST methods.

### DC method

**Fig. 1.**
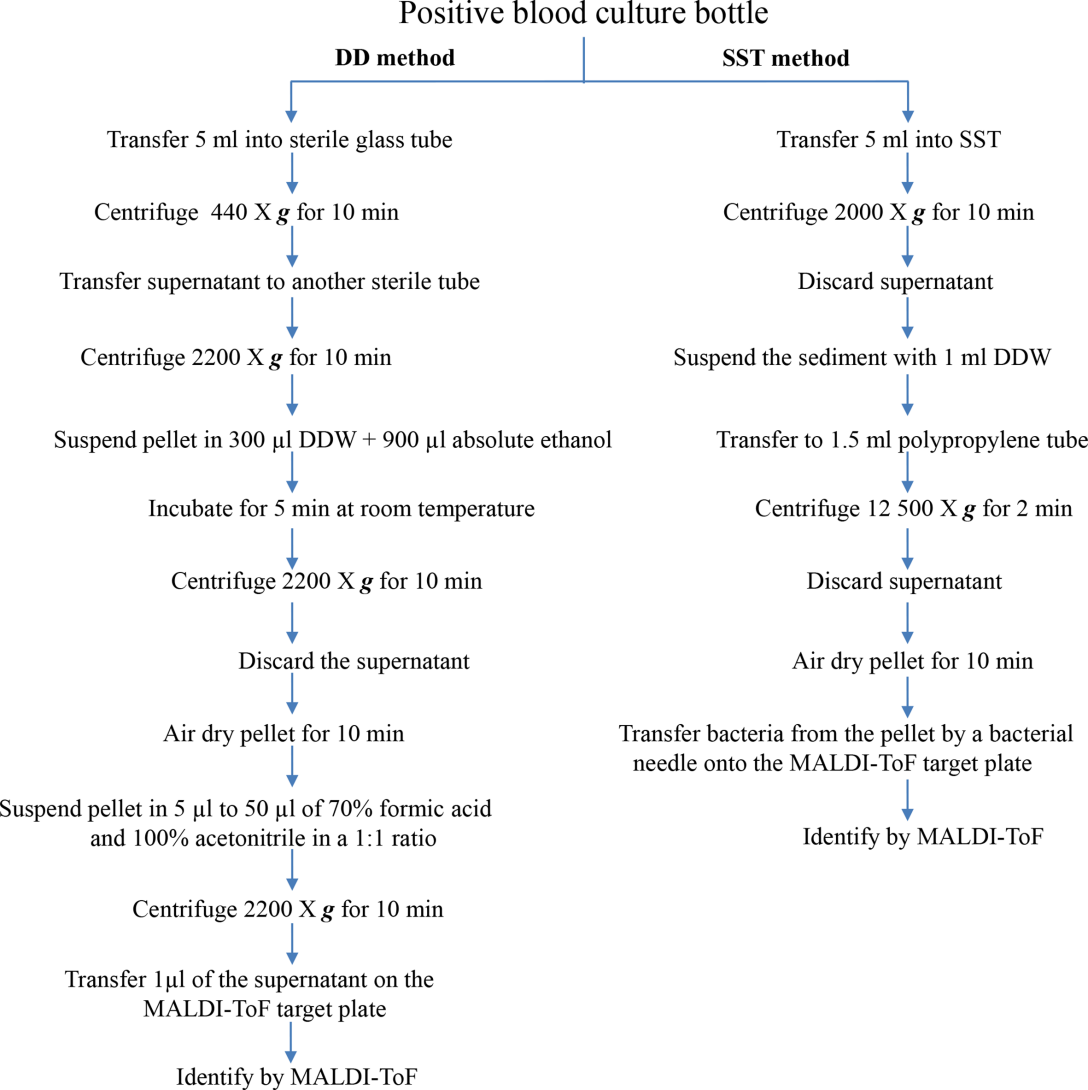
Comparison of workflow using DC vs SST sample preparation methods for micro-organism identification by MALDI-ToF.

One hundred positive blood culture samples were prepared as described previously [18–20, 25]. Briefly, 5 ml of the positive samples was transferred to sterile tubes and centrifuged at 440 ***g*** for 10 min. The supernatant was transferred to another sterile tube and centrifuged for 10 min at 2200 ***g***. The pellet was then suspended with 300 µl distilled water and 900 µl of absolute ethanol (Fluke, St Louis, MO, USA). The samples were incubated for 5 min at room temperature and centrifuged for 10 min at 2200 ***g***. The supernatant was removed and the pellet was dried for 10 min to remove residual ethanol. The pellet was resuspended in 5 µl to 50 µl of 70 % formic acid and 100 % acetonitrile (Carlo Reba, Milan, Italy) in a 1:1 ratio, according to the estimated pellet size. Following centrifugation for 10 min at 2200 ***g***, 1 µl of each supernatant was then spotted onto a sample spot of a MALDI target plate and allowed to dry (Fig. 1).

### SST method

One hundred blood culture samples were prepared by the SST method as previously described, with slight modifications [28]. Briefly, 5 ml from the culture bottles as transferred to SSTs. The tubes were centrifuged at 2000 ***g*** for 10 min, after which the bacteria sedimented on the surface of the silicon layer. Serum was discarded and the sediment was resuspended with 1 ml distilled water and transferred to a polypropylene tube (Eppendorf, Hamburg, Germany) and centrifuged at 12 500 ***g*** for 2 min. The supernatant was discarded and the bacterial pellet was dried at room temperature. Next, he bacteria from the pellet were transferred by loop onto the steel target plate for MALDI-ToF MS identification (Fig. 1).

To improve identification, Gram-positive bacteria on the target were overlaid with 0.6 µl absolute ethanol (Fluke), 0.6 µl formic acid (70 % v/v; Fluke) and acetonitrile (Carlo Reba) consecutively, with a few seconds’ drying interval between each reagent. After drying, the preparation was ready for MALDI-ToF MS identification.

### MALDI-ToF MS system identification and analysis

Identification of the micro-organisms by the MALDI-ToF MS technique was performed as described previously [28]. Briefly, 1 µl of α-cyano-4-hydroxycinnamic acid (HCCA) matrix was added to each spotted sample on the MALDI-ToF MS target and allowed to dry. The plate was then placed in the mass spectrometer for identification with Microflex and Compass software. Organisms were identified at the genus and species level [29].

### Statistical analysis

Descriptive statistics are presented in terms of mean, standard deviation and ranges. The *t*-test was used for differences in total score between the CD and SST groups. Fisher’s exact test was used to evaluate differences in the agreement rates between the CD and SST groups. *P*<0.05 was considered significant. SPSS version 21 was used for statistical analyses.

### Ethical approval

All specimens were collected as part of the routine clinical management of patients, according to national and local guidelines. Consequently, informed consent was not sought and approval from the Institutional Ethics Committee was not required.

## Results

Two hundred blood culture samples were included in this study. All isolates were tested by MALDI-ToF MS and conventional methods. Twenty-one isolates were identified by ChromAgar plates and 179 were identified by VITEK II. Among 100 samples prepared by the DC method, 75 % were correctly identified by MALDI-ToF MS, while 90 % of the 100 samples prepared by the SST method were identified correctly (*P*=0.0085). Identification of Gram-positive bacteria by MALDI-ToF MS versus the conventional method is shown in Table 1. Forty-seven samples were identified as Gram-positive bacteria, 28 were identified as Gram-negative bacteria and 25 were not identified when prepared by the DC method. Using the SST method, 50 samples were identified as Gram-positive bacteria, 40 as Gram-negative and 10 were not identified. These results demonstrate an accuracy of 100 % in Gram-negative identification by both methods, while the SST method is superior to the DC method in Gram-positive identification (85 % vs 68 %, respectively).

**Table 1.** Microbial identification by MALDI-ToF MS conventional protocol of samples prepared by DC method versus SST method

Micro-organism	No. isolates prepared with the DC method	No. isolates prepared with the SST method
Gram-positive	47	50
Gram-negative	28	40
No organism identified	25	10

DC, differential centrifugation SST, serum separator tube.

The Gram-positive bacteria identified by MALDI-ToF MS versus the conventional method are shown in Table 2. Following preparation by both methods, DC and SST, the majority of isolates were identified as coagulase-negative staphylococci (55 and 32 %, respectively). *Staphylococcus aureus* isolates were more abundant in the SST than the DC preparation, and *Streptococci* species, including *Enterococcus faecalis *, were more abundant in the SD DC preparation (2 *Streptococcus* *piscifermentans,* 12 *Streptococcus epidermidis*, 8 *Streptococcus* *hominids,* 1 *Streptococcus saccharolyticus *, 3 *Streptococcus* *haemolyticus* and 3 *Streptococcus* *capitis*). Three isolates were *Micrococcus luteus* and six were *S. aureus *. Nine isolates were identified as *Streptococci*, including two *Streptococcus pyogenes* and seven *Enterococcus faecalis *. These isolates were identified after preparation by the DC method.

**Table 2.** Gram-positive identification by MALDI-ToF MS with samples prepared by DC and SST versus conventional method

Blood cultures sample prepared with the DC method		Blood cultures sample prepared with the SST method	
Micro-organism	Samples identified correctly by MALDI-ToF MS and conventional methods	Micro-organism	Samples identified correctly by MALDI-ToF MS and conventional methods
Gram-positive	47	Gram-positive	50
Staphylococci species	35	Staphylococci species	41
* S. epidermidis *	12	* S. epidermidis *	12
* S. hominis *	8	* S. hominis *	6
* S. haemolyticus *	3	* S. haemolyticus *	1
* S. piscifermentans *	2	* S. pettenkoferi *	1
* S. saccharolyticus *	1	* S. capitis *	1
* S. capitis *	3	* S. aureus *	20
* S. aureus *	6	* Micrococcus luteus *	5
* M. luteus *	3	Streptococci species	4
Streptococci species	9	*Strep. salivarus*	1
* Strep. pyogenes *	2	* Enterococcus faecalis *	3
* E. faecalis *	7		

DC, differential centrifugation SST, serum separator tube.

All isolates prepared by DC or SST had confidence interval scores between 1.71–2.2 and 1.71–2.3, respectively (*P*=NS). The results show that all isolates shown in Table 2 identified by MALDI-ToF MS were in concordance with the final identification by the conventional method.

It is important to note that the MALDI-ToF MS system was unable to identify 25 samples prepared by the DC method and 10 samples prepared by the SST method (Table 3). Most of the isolates prepared by the DC method were Gram-positive, i.e. 16 *Staphylococci*, 6 *Streptococci*, 2 *Diphtheroides* spp. and 1 *Candida albicans,* which were identified by the conventional method. In parallel, the 10 samples that were not identified and had been prepared by the SST method were found to be *Staphylococci* species and *Streptococci,* as identified by the conventional method.

**Table 3.** The distribution of the bacteria reported by MALDI-ToF MS as ‘No organism identification possible’

Blood culture samples prepared by the DC method		Blood culture samples prepared by the SST method	
Micro-organism	Samples unidentified by MALDI-ToF MS and identified by conventional methods	Micro-organism	Samples unidentified by MALDI-ToF MS and identified by conventional methods
‘No organism identification possible’	25	‘No organism identification possible’	10
**Staphylococci species**	16	**Staphylococci species**	5
Coagulase-negative staphylococci	14	Coagulase-negative staphylococci	3
* S. aureus *	2	* S. aureus *	2
**Streptococci species**	6	**Streptococci species**	4
* Streptococcus vestibularis *	1	* Streptococcus gallolyticus *	2
Viridans streptococcus group	1	* E. faecalis *	2
* Streptococcus constellatus *	1	*Candida parapsilosis*	1
Streptococcus group G	1		
Streptococcus roup F	1		
*Anaerobic streptococcus*	1		
*Diphtheroides* spp.	2		
*C. albicans*	1		

The Gram-negative bacteria identified by MALDI-ToF MS versus the conventional method are shown in Table 4. Of the isolates prepared using the DC method, 28 were identified as Gram-negative. By contrast, of the bacteria prepared using the SST method, all 40 Gram-negative isolates tested were identified and the results were the same for both MALDI-ToF MS and the conventional method.

**Table 4.** Gram-negative identification by MALDI-ToF MS versus conventional method

Samples prepared by the DC method		Samples prepared by the SST method	
Micro-organism	Samples identified correctly by MALDI-ToF MS and conventional methods	Micro-organism	Samples identified correctly by MALDI-ToF MS and conventional methods
Gram-negative species	28	Gram negative species	40
* Escherichia coli *	9	* E. coli *	24
* Klebsiella pneumoniae *	3	* K. pneumoniae *	4
* Pseudomonas aeruginosa *	5	* P. aeruginosa *	2
* Enterobacter cloacae *	1	* E. cloacae *	2
* Citrobacter koseri *	1	* C. koseri *	4
* Enterobacter aeruginosa*	1	* Proteus mirabilis *	3
* Acinetobacter schindleri *	1	* Serratia marcescens *	1
* Acinetobacter baummannii*	1		
* Exiguobacterium aurantiacu*	1		
* Pseudomonas luteola *	1		
* Salmonella* spp.	1		
* Pasteurella multocida *	1		
* Bacteroides fragilis *	2		

## Discussion

The MALDI-ToF MS technology was originally developed to identify micro-organisms from isolated colonies. Successful attempts led to the use of this methodology to identify micro-organisms directly from positive blood cultures [28, 30]. The Gram stain of a positive blood culture, which is used routinely, provides data for managing empirical antimicrobial treatment of BSIs. MALDI-ToF MS for microbial identification provides more information than Gram staining and the isolate species is identified rapidly. Rapid identification of blood culture contaminants may also enable more rapid discontinuation of unnecessary antimicrobial therapy [30, 31]. Our laboratory adapted rapid identification of *Staphylococcus* spp. to determine the features of *S. aureus* with respect to the properties of methicillin-resistant *S. aureus* (MRSA) using molecular diagnosis by GenExpert [32]. Thus, we can quickly determine whether *S. aureus* is MRSA or not. This information enables the clinician to choose the appropriate antibiotic against *S. aureus *.

The present study compared the DC and SST methods for sample preparation and showed that 75 and 90 %, respectively, of positive blood cultures were identified correctly with MALDI-ToF MS technology when compared to the conventional procedure. We demonstrated that only 65.3 % of the Gram-positive bacteria tested were identified by MALDI-ToF MS following DC preparation, while 83.3 % were identified correctly using the SST method. A previous study [8] that used SST for MALDI-ToF MS identification reported that 73 % of Gram-positive bacteria were identified correctly. Our findings for the SST method are better, with 83.3 % correct identification of Gram-positive bacteria and 90 % correct identification among all the isolates tested. Regarding the DC method, our results showed 65.3 % correct identification of Gram-positive bacteria and 75 % correct identification among all isolates. These findings agree with those of Stevenson *et al.* [33], who found 76.4 % correct identification, and Juiz *et al*., who reported 77.7 % correct identification [19]. To the best of our knowledge, this study compared both methods for the first time and found that SST identifies Gram-positive bacteria more efficiently than DC. Moreover, the time required for sample preparation when using the SST method is shorter than that with DC (12–15 min vs 30 min), which is preferable.

Regarding the identification of Gram-negative bacteria, the agreement between MALDI-ToF MS and the conventional method was very high, whether the blood culture sample was prepared using the DC method or the SST method. This is true even though one isolate of *Salmonella* species was identified to the genus level and not to the species. The species was determined by an agglutination test with specific antibodies to various serotypes of the *Salmonella* species. Another isolate was correctly identified by MALDI-ToF MS and the conventional method at the level of the genus *Acinetobacter *, but differed in the species. MALDI-ToF MS identified it as *Schindleri* and the conventional method identified it as *lwoffii*. This disagreement in species identification might have occurred because the database for the conventional method did not include the species S*chindleri*. A similar discrepancy occurred with respect to the *Exiguobacterium aurantiacum* isolate*,* which was identified by MALDI-ToF MS but not by the conventional method. In these cases, 16S PCR and reference laboratory sequencing were used for final identification [34].

All the isolates in our study that resulted in ‘no organism identification possible’ by MALDI-ToF MS were identified as Gram-positive bacteria by the conventional method. This finding is in agreement with other studies [12, 14, 20, 33]. The inability of MALDI-ToF MS to identify a high percentage of Gram-positive bacteria may be related to the cell wall properties of these Gram-positive bacteria [21]. Some authors have suggested disrupting the peptidoglycan layer of the Gram-positive bacterial cell walls with ultrasound [13]. Other factors, such as low organism count may cause misidentification [35]. This could be overcome by concentrating the bacteria.

We note that one isolate was yeast and two were diphtheroid species, which were not identified by MALDI-ToF MS and were identified by the conventional method. The difficulty in identifying yeast may also be due to cell wall properties, as noted in another report [6]. Additional research is needed to improve blood culture sample preparation and increase the percentage of correctly identified Gram-positive bacteria and the small number of yeasts.

In conclusion, the use of the SST method to prepare blood culture samples for direct identification by MALDI-ToF MS is accurate and rapid. It provides a short turnaround time for the identification of micro-organisms isolated from blood cultures. This enables physicians to apply the appropriate antimicrobial treatment strategy and can help prevent unnecessary use of wide-spectrum antibiotics.
